# Supplementary material for: Electron correlation and relativistic effects in the excited states of radium monofluoride
Source: Nat Commun. 2025 Mar 3;16:2139. doi: 10.1038/s41467-025-55977-w (PMC11876649; doi:10.1038/s41467-025-55977-w)
Supplement: Supplementary file 1 — Supplementary Information [file 41467_2025_55977_MOESM1_ESM.pdf]

Supplementary Information: Electron correlation and relativistic effects in the excited states of radium monofluoride

M. Athanasakis-Kaklamanakis,[1,2,3,*] S. G. Wilkins,[4,5,†] L. V. Skripnikov,[6,‡] Á. Koszorús,[1,2] A. A. Breier,[7,8] O. Ahmad,[2] M. Au,[9,10] S. W. Bai,[11] I. Belošević,[12] J. Berbalk,[2] R. Berger,[13] C. Bernerd,[9] M. L. Bissell,[14] A. Borschevsky,[15] A. Brinson,[4] K. Chrysalidis,[9] T. E. Cocolios,[2] R. P. de Groote,[2] A. Dorne,[2] C. M. Fajardo-Zambrano,[2] R. W. Field,[16] K. T. Flanagan,[14,17] S. Franchoo,[18,19] R. F. Garcia Ruiz,[4,5] K. Gaul,[13] S. Geldhof,[2] T. F. Giesen,[8] D. Hanstorp,[20] R. Heinke,[9] P. Imgram,[2] T. A. Isaev,[6] A. A. Kyuberis,[15] S. Kujanpää,[21] L. Lalanne,[2,1] P. Lassègues,[2] J. Lim,[3] Y. C. Liu,[11] K. M. Lynch,[14] A. McGlone,[14] W. C. Mei,[11] G. Neyens,[2,§] M. Nichols,[20] L. Nies,[1] L. F. Pašteka,[15,22] H. A. Perrett,[14] A. Raggio,[21] J. R. Reilly,[14] S. Rothe,[9] E. Smets,[2] S.-M. Udrescu,[4] B. van den Borne,[2] Q. Wang,[23] J. Warbinek,[24,25] J. Wessolek,[9,14] X. F. Yang,[11] and C. Zülch[13]

[1]*Experimental Physics Department, CERN, CH-1211 Geneva 23, Switzerland*
[2]*KU Leuven, Instituut voor Kern- en Stralingsfysica, B-3001 Leuven, Belgium*
[3]*Blackett Laboratory, Centre for Cold Matter, Imperial College London, SW7 2AZ London, United Kingdom*
[4]*Department of Physics, Massachusetts Institute of Technology, Cambridge, MA 02139, USA*
[5]*Laboratory for Nuclear Science, Massachusetts Institute of Technology, Cambridge, MA 02139, USA*
[6]*Affiliated with an institute covered by a cooperation agreement with CERN.*
[7]*Institut für Optik und Atomare Physik, Technische Universität Berlin, 10623 Berlin, Germany*
[8]*Laboratory for Astrophysics, Institute of Physics, University of Kassel, Kassel 34132, Germany*
[9]*Systems Department, CERN, CH-1211 Geneva 23, Switzerland*
[10]*Department of Chemistry, Johannes Gutenberg-Universität Mainz, 55099 Mainz, Germany*
[11]*School of Physics and State Key Laboratory of Nuclear Physics and Technology, Peking University, Beijing 100971, China*
[12]*TRIUMF, Vancouver BC V6T 2A3, Canada*
[13]*Fachbereich Chemie, Philipps-Universität Marburg, Marburg 35032, Germany*
[14]*Department of Physics and Astronomy, The University of Manchester, Manchester M13 9PL, United Kingdom*
[15]*Van Swinderen Institute of Particle Physics and Gravity, University of Groningen, Groningen 9712 CP, Netherlands*
[16]*Department of Chemistry, Massachusetts Institute of Technology, Cambridge, MA 02139, USA*
[17]*Photon Science Institute, The University of Manchester, Manchester M13 9PY, United Kingdom*
[18]*Laboratoire Irène Joliot-Curie, Orsay F-91405, France*
[19]*University Paris-Saclay, Orsay F-91405, France*
[20]*Department of Physics, University of Gothenburg, Gothenburg SE-41296, Sweden*
[21]*Department of Physics, University of Jyväskylä, Jyväskylä FI-40014, Finland*
[22]*Department of Physical and Theoretical Chemistry, Faculty of Natural Sciences, Comenius University, Bratislava, Slovakia*
[23]*School of Nuclear Science and Technology, Lanzhou University, Lanzhou 730000, China*
[24]*GSI Helmholtzzentrum für Schwerionenforschung GmbH, 64291 Darmstadt, Germany*
[25]*Department of Chemistry - TRIGA Site, Johannes Gutenberg-Universität Mainz, 55128 Mainz, Germany*

SUPPLEMENTARY NOTE 1: EXTENDED METHODS

Data analysis

The measured wavenumbers in the acquired spectra were firstly Doppler-shifted to the molecular rest frame wavenumber $\tilde{\nu}$ according to the expression $\tilde{\nu} = \frac{1-\beta}{\sqrt{1-\beta^2}}\tilde{\nu}_0$, where $\beta = v/c$ with c the speed of light, and $\tilde{\nu}_0$ the wavenumber in the lab frame. The velocity of the beam was determined from the ion kinetic energy, which was defined by the platform voltage of the radiofrequency cooler-buncher that drifted over time between 39,905 and 39,910 V. Fluctuations and drifts of the platform voltage were monitored by continuous measurements (1 measurement per second) of the real voltage using a calibrated potential divider (PTB PT60-2) and a digital multimeter (Agilent 34401A). The voltage measurements (precision of 10 mV at 40 kV) were then used to accurately determine the velocity of the ^{226}RaF beam for each wavenumber measurement. Following Doppler-shifting, the spectra obtained with fundamental Ti:Sa (∼3 GHz) and the narrower (∼0.8 GHz) Cobra dye laser were binned into a histogram with a bin size of 3 GHz, and the spectra obtained with the broader (∼9 GHz) Spectra Physics dye laser and second-harmonic Ti:Sa (∼6 GHz) with a bin size of 9 GHz.

The binned spectra were analyzed using the contour-

* m.athkak@cern.ch
† wilkinss@mit.edu
‡ skripnikov_lv@pnpi.nrcki.ru
§ gerda.neyens@kuleuven.be

fitting routine of the PGOPHER package using the effective Hamiltonian for linear molecules embedded in PGOPHER [1]; for $^2\Sigma$ states, the molecular constants relevant to Hund's case b were used, and for $^2\Pi$ and $^2\Delta$ states, the constants for Hund's case a were used. The state origin T_0, rotational constant B, spin-rotation coupling constant γ (only for $^2\Sigma$ states), and Λ-doubling constant p (only for $^2\Pi$ states) were varied during the fitting routine to reach agreement between simulation and experiment.

All measured spectra involved electronic transitions starting from either the $X\ ^2\Sigma_{1/2}$ or the $A\ ^2\Pi_{1/2}$ states. The molecular parameters of these two states are known from the rotationally resolved ($\Delta f \approx 100$ MHz) laser spectroscopy of the $A\ ^2\Pi_{1/2} \leftarrow X\ ^2\Sigma_{1/2}$ transition in ^{226}RaF [2]. As a result, in the present study only the properties of the upper vibronic state in each spectrum were varied during the fitting procedure.

The statistical uncertainty in the excitation energies was extracted by the standard deviation of the fitted parameter given by the contour-fitting routine of PGOPHER. The uncertainty in the raw data was the error in the count rate (y-axis) in the spectra, which was determined as the square root of the number of data points in each bin. The standard deviations of the fitted excitation energies were scaled by the square root of the reduced chi-squared of the fit $\sqrt{\chi^2_r}$. These correspond to the denoted statistical errors are denoted in the main text.

A systematic uncertainty is considered for all excitation energies, which corresponds to the Voigt-peak linewidth set in PGOPHER to best match the observed linewidths for each spectrum, and aims to account for the propensity of the contour-fitting routine to converge to local minima, as it cannot move a simulated line by more than the set linewidth [1]. An additional component of 0.02 cm^{-1} is added to account for the combined sources of systematic error stemming from the experimental equipment (the sources of this error are discussed in Ref. [2]).

The best-fit values and uncertainties of the excitation energies were also obtained independently using a chi-squared minimization code written in Python and the correlated errors were determined by inspecting the corner plots of the fitted parameters. The results of the independent fitting were consistent with the results from the PGOPHER analysis, and thus the latter are used here.

The resolution of the spectra of transitions to the $G\ ^2\Pi_{1/2}$ and $E\ ^2\Sigma_{1/2}$ states was high enough to enable an analysis using the line-fitting routine of PGOPHER, which was found to yield results in agreement within 1σ with contour-fitting of the same spectra. Only contour-fitting results are included in this work for consistency.

Spin-orbit constants

The molecular spin-orbit (SO) constants A were extracted as:

$$A = \frac{1}{2S\Lambda}\left(E_{\Lambda+S} - E_{\Lambda-S}\right) \tag{1}$$

All electronic states in alkaline-earth monofluorides have a single unpaired valence electron, and thus $S = 1/2$. For $^2\Pi$ states, $\Lambda = 1$, while for $^2\Delta$ states, $\Lambda = 2$. For the SO constants in RaF, the observed excitation energies reported in Table 1 of the main text were used.

For the microscopic SO constants ζ in Ra$^+$, the excitation energies in the NIST Atomic Spectra Database were used. For ^2P states:

$$\zeta = \frac{2}{3}\left(E_{L+S} - E_{L-S}\right) \tag{2}$$

and for ^2D states:

$$\zeta = \frac{2}{5}\left(E_{L+S} - E_{L-S}\right) \tag{3}$$

State assignment

The following subsections provide details that guided the assignment of term symbols for the observed spectra. In addition to the specifics of each spectrum, the assignment was guided by the agreement between the experimentally determined rotational constant of the $v = 0$ state $B_{0,\mathrm{obs}}$ and the theoretically determined equilibrium rotational constant $B_{\mathrm{e,th}}$ across all states. While $B_{0,\mathrm{obs}}$ and $B_{\mathrm{e,th}}$ are different molecular constants, they are closely related and at the given level of precision a large deviation between the two would indicate an incorrect assignment.

$A\ ^2\Pi_{3/2}$

The spectrum of the transition from the electronic ground state to $A\ ^2\Pi_{3/2}$ was measured in the region where it had been previously measured in Ref. [3]. The spectrum was analyzed in this work using PGOPHER and the excitation energy was extracted from the molecular Hamiltonian, leading to a small correction and reassignment compared to the previous result reported in Ref. [3]. The measured spectrum was congested, with the contours of the vibrational transitions being largely overlapped, which could explain the deviation of the observed vibrational spacing ω_{obs} from ω_{th} in Supplementary Table V.

$B\ ^2\Delta_{3/2}$

The excitation energy of the $B\ ^2\Delta_{3/2}$ state was assigned via the spectrum of a transition from the $X\ ^2\Sigma_{1/2}$ electronic ground state, where a vibrational progression was observed and interpreted as belonging to the diagonal transitions $\Delta v = 0$ with $v'' = 0 - 3$. The observed excitation energy of $14{,}333.00(161)_{\mathrm{stat}}(51)_{\mathrm{syst}}$ cm^{-1} and the observed vibrational spacing of $\omega_{\mathrm{obs}} = 425.9(34)$ cm^{-1} are in excellent agreement with the calculated values of 14,300(61) cm^{-1} and $\omega_{\mathrm{th}} = 426(4)$ cm^{-1}, respectively, for the $B\ ^2\Delta_{3/2}$ state, while the theoretical values disagree with the previous tentative assignment in Ref. [3]. As a result, the new assignment for the excitation energy of $B\ ^2\Delta_{3/2}$ is adopted instead of the previous tentative assignment at 15,142.7(5) cm^{-1} from Ref. [3].

$B\ ^2\Delta_{5/2}$

The previously measured [3] spectrum that was tentatively assigned as a transition to $B\ ^2\Delta_{3/2}$ is now tentatively reassigned as a transition to $B\ ^2\Delta_{5/2}$ and analyzed using PGOPHER, extracting both the excitation energy and the vibrational spacing.

The observed excitation energy is in agreement with the theoretical prediction for the $B\ ^2\Delta_{5/2}$ state and the observed vibrational spacing of $\omega_{\mathrm{obs}} = 428.6(11)$ cm^{-1} is also in good agreement with $\omega_{\mathrm{th}} = 430(4)$ cm^{-1} (Supplementary Table V).

The transition $B\ ^2\Delta_{5/2} \leftarrow X\ ^2\Sigma_{1/2}$ is expected to be dipole-forbidden (due to $\Delta\Lambda = 2$ and $\Delta\Omega = 2$) but allowed by the L-uncoupling interaction [4] of the $B\ ^2\Delta_{5/2}$ state with $A\ ^2\Pi_{3/2}$. In the absence of additional information that can be used to unambiguously identify the upper state, this assignment remains tentative.

$C\ ^2\Sigma_{1/2}$

The new assignment for the energy of the $C\ ^2\Sigma_{1/2}$ state follows from the observation of a transition from the electronic ground state at $16{,}612.06(18)_{\mathrm{stat}}(51)_{\mathrm{syst}}$ cm^{-1}, in close agreement with the prediction at 16,615(69) cm^{-1}. This transition lies outside the range that was scanned in Ref. [3] and was thus not previously observed. The spectral profile of this transition is very similar to that of the transition at 16,175.2 cm^{-1} that was previously assigned as $C\ ^2\Sigma_{1/2} \leftarrow X\ ^2\Sigma_{1/2}$ ($v' = 0 \leftarrow v'' = 0$), but its intensity is twice that of the transition at 16,175.2 cm^{-1}. As a result, it is concluded that it is the newly discovered transition that in fact corresponds to $C\ ^2\Sigma_{1/2} \leftarrow X\ ^2\Sigma_{1/2}$ ($v' = 0 \leftarrow v'' = 0$) and the previously identified transition corresponds to ($v' = 0 \leftarrow v'' = 1$).

$D\ ^2\Pi_{1/2}$ *and* $D\ ^2\Pi_{3/2}$

The excitation energies of the $D\ ^2\Pi_{1/2}$ and $D\ ^2\Pi_{3/2}$ states were assigned based on transitions starting from the electronic ground state $X\ ^2\Sigma_{1/2}$.

For each state, the diagonal vibrational progression of the transition from the ground state was observed up to $v' = 2 \leftarrow v'' = 2$. This allowed the extraction of the vibrational spacing for the two states, giving an observed value of $\omega_{\mathrm{obs}} = 431.1(4)$ cm^{-1} for $D\ ^2\Pi_{1/2}$ as opposed to the calculated value of $\omega_{\mathrm{th}} = 434(4)$ cm^{-1}, and $\omega_{\mathrm{obs}} = 441.8(8)$ cm^{-1} for $D\ ^2\Pi_{3/2}$ as opposed to $\omega_{\mathrm{th}} = 444(4)$ cm^{-1} (Supplementary Table V). The agreement with theory in terms of magnitude as well as observing ω to be larger for the $D\ ^2\Pi_{3/2}$ state (and larger than for the ground state) support the assignment in this work. The contours of the spectra interpreted as corresponding to transitions to the $D\ ^2\Pi_{1/2}$ vibrational states are distinctly different from the spectra interpreted as corresponding to the $D\ ^2\Pi_{3/2}$ states. Therefore, the possibility of the two sets of spectra corresponding to one diagonal and one non-diagonal vibrational progressions ($\Delta v = 0$ and $\Delta v = \pm 1$) to the same upper electronic state is excluded.

The spectrum of the transition to the $D\ ^2\Pi_{1/2}$ state was highly congested with the contours of the vibrational transitions largely overlapping, so a contribution from $v' = 3 \leftarrow v'' = 3$ was also taken into account as its presence could not be excluded. This contribution affected the results of the $v' = 0$ state within uncertainties.

As a validity check for the assignment of $D\ ^2\Pi_{1/2}$ without the use of the theoretical calculations, the Λ-doubling constant p extracted from the contour fit (see Supplementary Table V) can be compared to the value extracted via the pure precession approximation for the interaction between the $^2\Pi$ state and neighboring $^2\Sigma$ states [5]:

$$p_{\mathrm{pure}} = 2AB_v l(l+1)/\Delta E_{\Pi\Sigma} \qquad (4)$$

Using $l = 1$, $B = 0.1922(4)$ (Supplementary Table V), and $A = 362(1)$, and considering interaction only with the neighboring $C\ ^2\Sigma_{1/2}$ state, the approximation results in $p_{\mathrm{pure}} = 0.049$. This value is in agreement with the value extracted from the contour fit (Supplementary Table V).

$E\ ^2\Sigma_{1/2}$

The energy of the $E\ ^2\Sigma_{1/2}$ state was assigned based on a transition starting from $A\ ^2\Pi_{1/2}$ ($v = 0$), as the first laser step was exciting the $A\ ^2\Pi_{1/2}$ ($v' = 0$) $\leftarrow X\ ^2\Sigma_{1/2}$ ($v'' = 0$) transition.

Only two transitions were discovered in a range of 1,500 cm^{-1} around the predicted excitation energy of the $E\ ^2\Sigma_{1/2}$ state. The spectra of both transitions

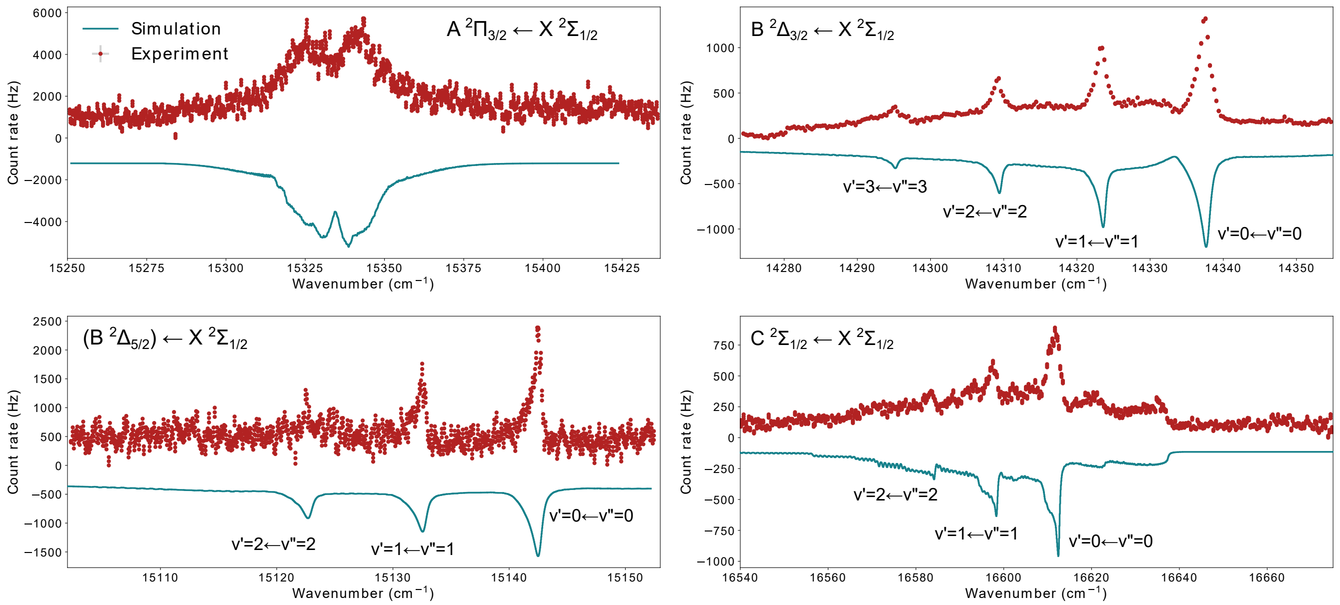

SUPPLEMENTARY FIGURE 1. Measured and simulated spectra for the transitions from the ground state to the low-lying states. The different vibrational bands are overlapped in the spectrum to $A\ ^2\Pi_{3/2}$, but are included in the simulation.

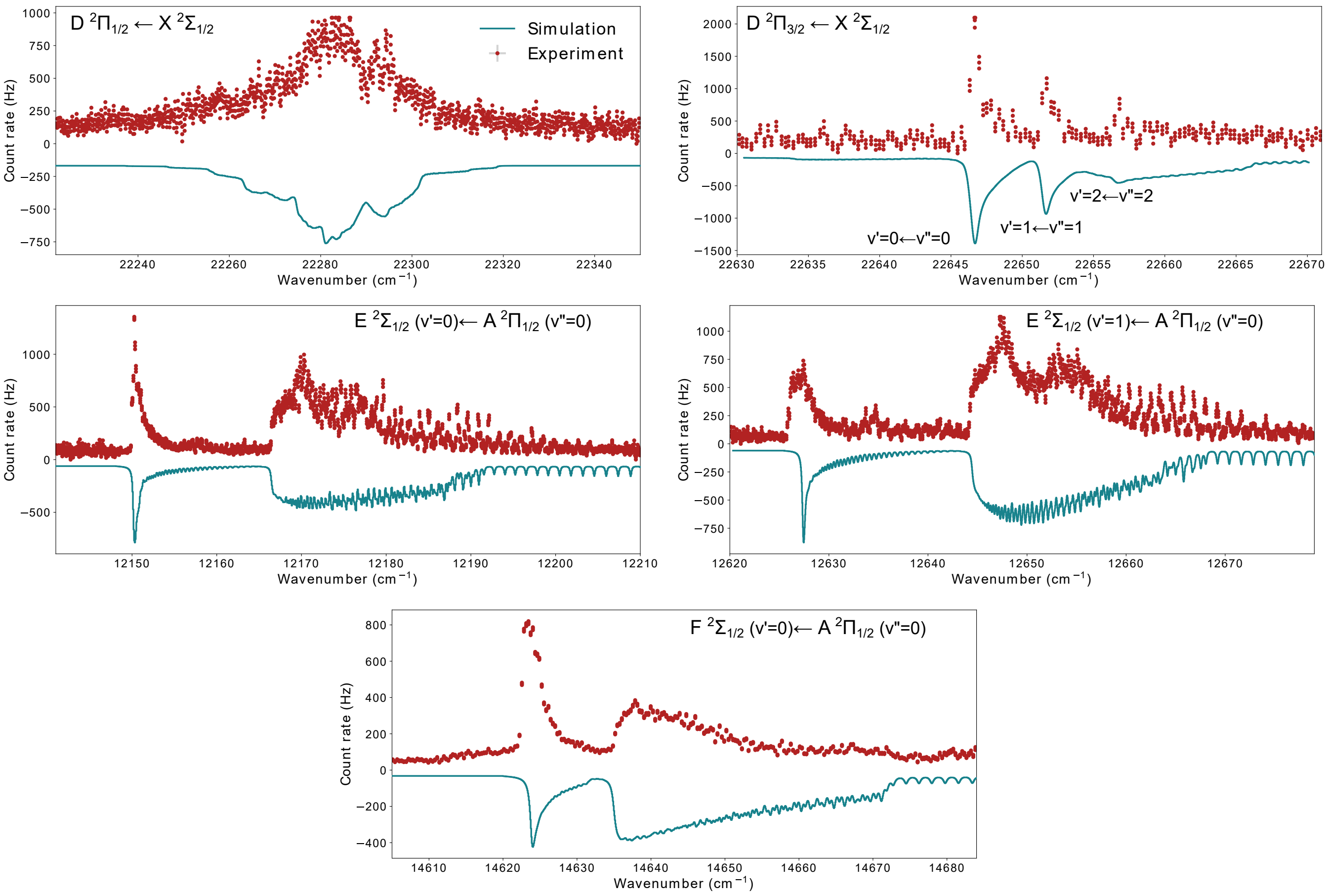

SUPPLEMENTARY FIGURE 2. Measured and simulated spectra for the transitions from the ground state to the low-lying states. The different vibrational bands are overlapped in the spectrum of $D\ ^2\Pi_{1/2} \leftarrow A\ ^2\Pi_{1/2}$, but are included in the simulation.

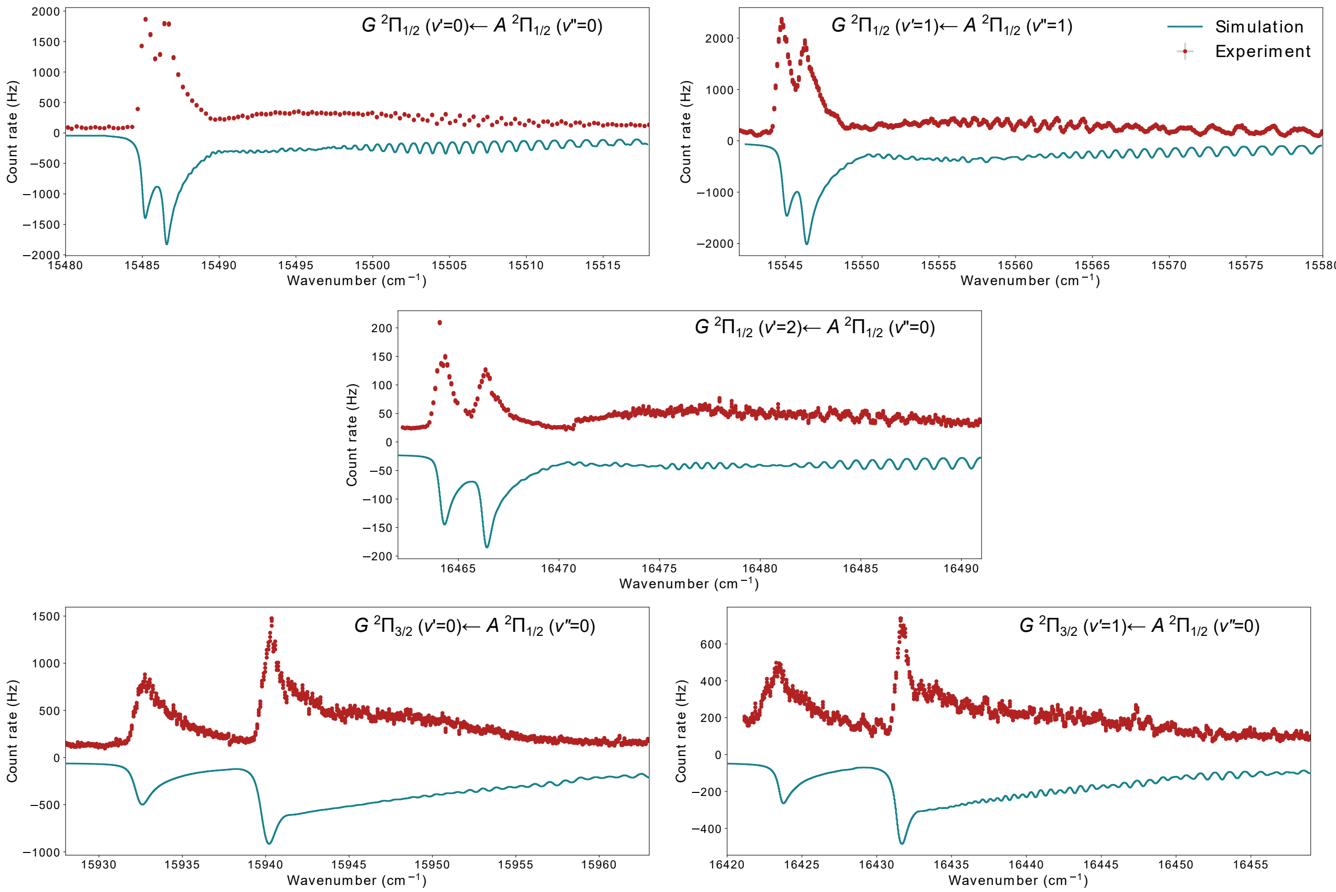

SUPPLEMENTARY FIGURE 3. Measured and simulated spectra of diagonal and off-diagonal vibronic transitions from $A\ ^2\Pi_{1/2}$ to the $G\ ^2\Pi$ states.

have very similar shapes and are separated by $\omega_{\rm obs} = 478.0(2)$ cm^{-1}, which is in close agreement with $\omega_{\rm th} = 481(5)$ cm^{-1} (Supplementary Table V). Since the transition starts from $v = 0$ of the lower state, the two measured spectra were interpreted as belonging to transitions to $v = 0$ and $v = 1$ of the $E\ ^2\Sigma_{1/2}$ state, which is the only state predicted to lie within a few thousand cm^{-1} of the measured structures.

It was observed that, for this spectrum in particular, using a Gaussian temperature distribution centered at room temperature improved the reduced chi-squared of the fit.

$F\ ^2\Sigma_{1/2}$

The energy of the $F\ ^2\Sigma_{1/2}$ state was assigned based on a transition starting from $A\ ^2\Pi_{1/2}$ ($v = 0$), as the first laser step was exciting the $A\ ^2\Pi_{1/2}$ ($v' = 0$) ← $X\ ^2\Sigma_{1/2}$ ($v'' = 0$) transition. Therefore, the spectrum was interpreted as $F\ ^2\Sigma_{1/2}$ ← $A\ ^2\Pi_{1/2}$ ($v' = 0 \leftarrow v'' = 0$). A second spectrum was obtained starting from $A\ ^2\Pi_{1/2}$ ($v = 1$), interpreted as $F\ ^2\Sigma_{1/2}$ ← $A\ ^2\Pi_{1/2}$ ($v' = 1 \leftarrow v'' = 1$).

Only one transition was discovered in a range of 500 cm^{-1} around the predicted excitation energy of the $F\ ^2\Sigma_{1/2}$ state, starting from the $A\ ^2\Pi_{1/2}$ $v = 0$ state. The contour of the spectrum resembled closely that of the transition to $E\ ^2\Sigma_{1/2}$ $v = 0$ indicating that the upper state has a similar wavefunction, as it is expected for the $F\ ^2\Sigma_{1/2}$ state. The high intensity of the measured signal excludes the possibility of the spectrum corresponding to a high overtone transition to a vibrational state of $E\ ^2\Sigma_{1/2}$ (such as $v' = 5 \leftarrow v'' = 0$). Therefore, this spectrum is attributed to $F\ ^2\Sigma_{1/2}$ $v = 0$ as the upper state and the excitation energy is assigned accordingly.

The observed vibrational spacing $\omega_{\rm obs} = 491.7(18)$ cm^{-1}, extracted using the $0 \leftarrow 0$ and $1 \leftarrow 1$ transition frequencies is in close agreement with the calculated $\omega_{\rm th} = 488(5)$ cm^{-1} (Supplementary Table V).

$G\ ^2\Pi_{1/2}$

The energy of the $G\ ^2\Pi_{1/2}$ state was assigned based on a transition starting from $A\ ^2\Pi_{1/2}$ ($v = 0$), as the first laser step was exciting the $A\ ^2\Pi_{1/2}$ ($v' = 0$) $\leftarrow X\ ^2\Sigma_{1/2}$ ($v'' = 0$) transition.

A spectrum that starts from the same lower state as the one assigned as $G\ ^2\Pi_{1/2} \leftarrow A\ ^2\Pi_{1/2}$ ($v' = 0 \leftarrow v'' = 0$) and has a very similar spectral profile was measured at approximately 980 cm^{-1} higher in energy (corresponding to approximately two times the calculated vibrational spacing for this state, Supplementary Table V), which was interpreted as a transition to $G\ ^2\Pi_{1/2}$ ($v = 2$). Additionally, in a transition starting from the $A\ ^2\Pi_{1/2}$ ($v = 1$) state, a spectrum was observed whose upper state was at an excitation energy half-way between those of the $v = 0$ and $v = 2$ states, with a very similar spectral profile. Therefore, this third spectrum was interpreted as belonging to a transition to $G\ ^2\Pi_{1/2}$ ($v = 1$). The only other predicted state in the vicinity of $G\ ^2\Pi_{1/2}$ is $G\ ^2\Pi_{3/2}$. The transition strength of $G\ ^2\Pi_{1/2} \leftarrow A\ ^2\Pi_{1/2}$ ($v' = 0 \leftarrow v'' = 0$) was the largest among all transitions measured from $A\ ^2\Pi_{1/2}$ in this experiment, which is highly improbable for the nominally forbidden transition $G\ ^2\Pi_{3/2} \leftarrow A\ ^2\Pi_{1/2}$. Therefore, the observed spectrum was assigned as $G\ ^2\Pi_{1/2} \leftarrow A\ ^2\Pi_{1/2}$ ($v' = 0 \leftarrow v'' = 0$).

Using the pure precession approximation, as done for $D\ ^2\Pi_{1/2}$, and assuming mixing only with the neighboring ($H\ ^2\Sigma_{1/2}$) state, $p_{\text{pure}} = -0.407$ is in good agreement with the fitted value $p_{\text{obs}} = -0.360(42)$.

$G\ ^2\Pi_{3/2}$

The energy of the $G\ ^2\Pi_{3/2}$ state was assigned based on a transition starting from $A\ ^2\Pi_{1/2}$ ($v = 0$), as the first laser step was exciting the $A\ ^2\Pi_{1/2}$ ($v' = 0$) $\leftarrow X\ ^2\Sigma_{1/2}$ ($v'' = 0$) transition.

Within a range of 400 cm^{-1} around the prediction for the transition energy to the $G\ ^2\Pi_{3/2}$ state, spectra of two transitions were found, and the excitation energy of both would be in agreement with the prediction.

However, for only one of the two transitions, a second spectrum also starting from $A\ ^2\Pi_{1/2}$ ($v = 0$) was measured at 491.6(4) cm^{-1} higher that has a very similar spectral profile. This wavenumber difference is in agreement with the calculated vibrational spacing for the $G\ ^2\Pi_{3/2}$ state (Supplementary Table V), and so these two spectra that had the same spectral profile were interpreted as transitions to $v = 0$ and $v = 1$ of $G\ ^2\Pi_{3/2}$.

$H\ ^2\Sigma_{1/2}$, $I\ ^2\Delta_{3/2}$, and $I\ ^2\Delta_{5/2}$

The energies of these three states were assigned based on transitions starting from $A\ ^2\Pi_{1/2}$ ($v = 0$), as the first

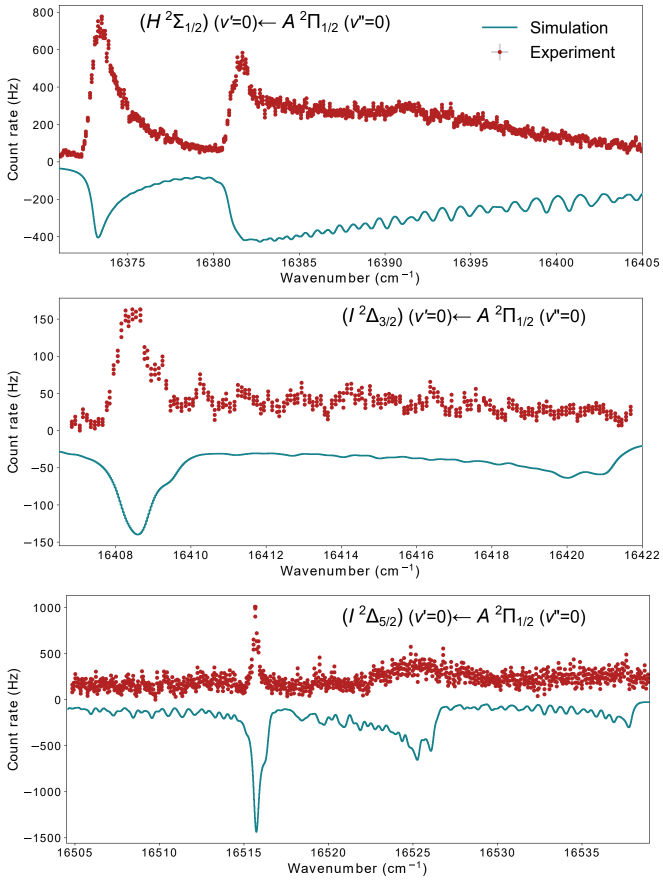

SUPPLEMENTARY FIGURE 4. Measured and simulated spectra from $A\ ^2\Pi_{1/2}$ to the tentatively assigned highest-lying states in this study.

laser step was exciting the $A\ ^2\Pi_{1/2}$ ($v' = 0$) $\leftarrow X\ ^2\Sigma_{1/2}$ ($v'' = 0$) transition in all cases.

In the vicinity of the transitions assigned to these three states, multiple transitions were observed. Two of those transitions were identified as the $v' = 1 \leftarrow v'' = 0$ and $v' = 2 \leftarrow v'' = 0$ transitions to the $G\ ^2\Pi_{3/2}$ and $G\ ^2\Pi_{1/2}$ states, respectively.

At approximately 16,175 cm^{-1}, the transition $C\ ^2\Sigma_{1/2} \leftarrow X\ ^2\Sigma_{1/2}$ ($v' = 0 \leftarrow v'' = 1$) was identified, while at approximately 16,610 cm^{-1}, the transition $C\ ^2\Sigma_{1/2} \leftarrow X\ ^2\Sigma_{1/2}$ ($v' = 0 \leftarrow v'' = 0$) was identified. These two transitions did not arise from the combined effect of all three lasers in the excitation scheme, but only from the scanning laser and the non-resonant ionization step. Therefore, they were identified as transitions from the electronic ground state, rather than from $A\ ^2\Pi_{1/2}$ ($v = 0$).

Two bands were assigned as transitions to $H\ ^2\Sigma_{1/2}$ ($v = 0$) and $I\ ^2\Delta_{3/2}$ ($v = 0$) based on the computational predictions. As the predicted energies of the two states are within uncertainties of each other, their assignment is tentative.

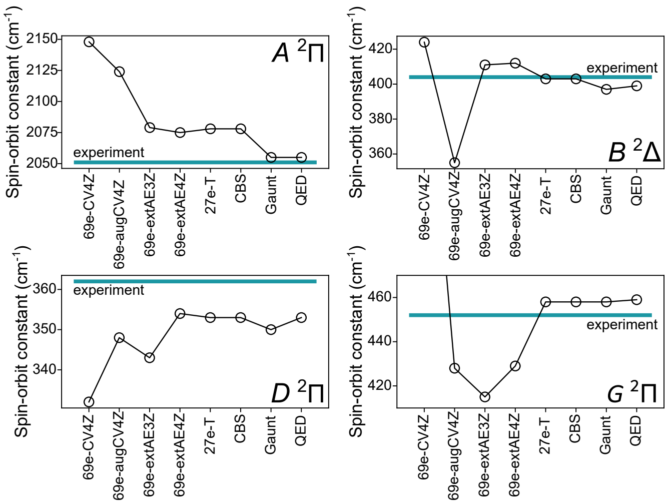

SUPPLEMENTARY FIGURE 5. Calculated and observed SO constants for states in RaF as a function of theoretical corrections.

The spectrum that was assigned as belonging to the transition to $I\ ^2\Delta_{5/2}$ was identified due to its significantly lower intensity compared to all other spectra, which is consistent with the dipole-forbidden $I\ ^2\Delta_{5/2} \leftarrow A\ ^2\Pi_{1/2}$ $(v' = 0 \leftarrow v'' = 0)$ transition. As the transition is dipole-forbidden due to $\Delta\Omega = 2$, the assignment is tentative.

One more, very weak spectral feature was identified at an excitation energy of approximately 29,650 cm^{-1}, whose spectral profile suggests the form of a $^2\Sigma \leftarrow ^2\Pi$ spectrum. Neither a firm nor a tentative assignment was possible at the present time. Based on the T_0 and $\omega_{\rm obs}$ for the $F\ ^2\Sigma_{1/2}$ state, this spectrum could correspond to an overtone transition of $F\ ^2\Sigma_{1/2} \leftarrow A\ ^2\Pi_{1/2}$ (such as $v' = 3 \leftarrow v'' = 0$), whose expected strength is consistent with the very low intensity of the observed structure. However, more information on the anharmonicity of the vibrational spacing of $F\ ^2\Sigma_{1/2}$ for higher values of v is required to make this assignment definite.

SUPPLEMENTARY TABLE I. Comparison of electronic excitation wavenumbers (T_0, in cm^{-1}) assigned to low-lying states in RaF between Ref. [3] and the present work. All assignments refer to the $v = 0$ vibrational state of each electronic state.

	Garcia et al. 2020	This work	Theory
$B\ ^2\Delta_{3/2}$	15,142.7(5)*	$14{,}333.00(161)_{\mathrm{stat}}(51)_{\mathrm{syst}}$	14,300(61)
$(B\ ^2\Delta_{5/2})$	—	$15{,}140.36(48)_{\mathrm{stat}}(51)_{\mathrm{syst}}$	15,099(70)
$A\ ^2\Pi_{3/2}$	15,344.6(50)	$15{,}335.73(49)_{\mathrm{stat}}(62)_{\mathrm{syst}}$	15,355(35)
$C\ ^2\Sigma_{1/2}$	16,175.2(5)$^{\$}$	$16{,}612.06(18)_{\mathrm{stat}}(51)_{\mathrm{syst}}$	16,615(69)

* Tentatively assigned in Ref. [3].
$^{\$}$ Following the new measurements, this transition is re-interpreted as $C\ ^2\Sigma_{1/2} \leftarrow X\ ^2\Sigma_{1/2}$ ($v' = 0 \leftarrow v'' = 1$). We place the $v' = 0 \leftarrow v'' = 0$ transition at $16{,}612.06(18)_{\mathrm{stat}}(51)_{\mathrm{syst}}\ \mathrm{cm}^{-1}$, in agreement with the theoretical prediction.

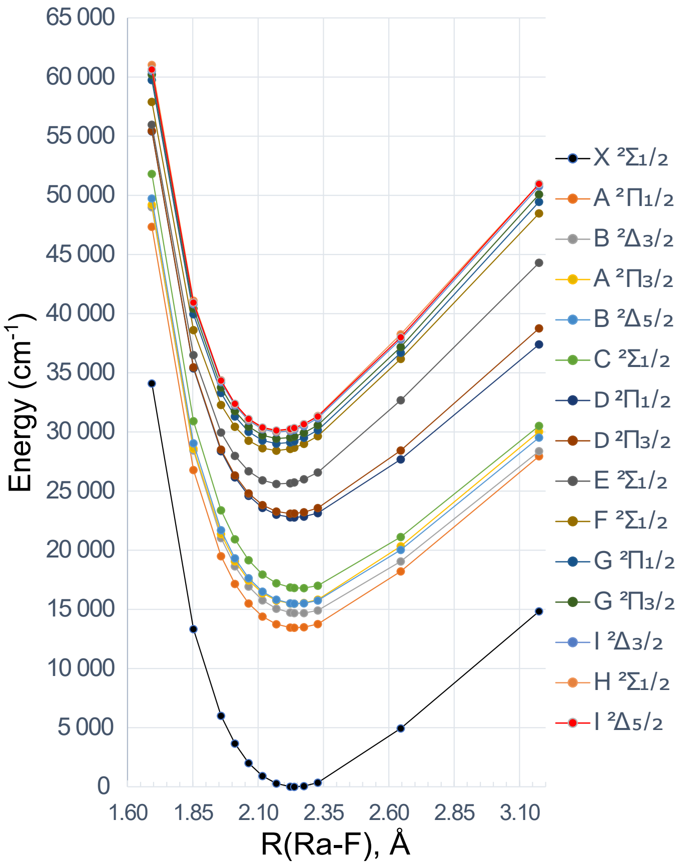

SUPPLEMENTARY FIGURE 6. Calculated potential energy curves of the RaF molecule for the electronic states studied in this work.

SUPPLEMENTARY TABLE II. Theoretical adiabatic electronic excitation energies (T_e, in cm^{-1}) calculated at the 69e-FS-RCCSD-extAE4Z level including higher-order corrections. Each column presents cumulative results, adding a contribution to the values in the column to its left. In the final column, the zero-point vibrational energy is added to T_e to arrive at T_0 that is compared with experiment.

State	69e-extAE4Z	+27e-CCSDT	+CBS	+Gaunt	+QED	+ZPE (T_0)
$X\ ^2\Sigma_{1/2}$	0	0	0	0	0	0
$A\ ^2\Pi_{1/2}$	13,406	13,360	13,354	13,359	13,301	13,299
$B\ ^2\Delta_{3/2}$	14,582	14,531	14,444	14,379	14,307	14,300
$B\ ^2\Delta_{5/2}$	15,403	15,336	15,249	15,171	15,103	15,099
$A\ ^2\Pi_{3/2}$	15,481	15,438	15,432	15,414	15,357	15,355
$C\ ^2\Sigma_{1/2}$	16,785	16,694	16,685	16,674	16,622	16,615
$D\ ^2\Pi_{1/2}$	22,724	22,504	22,442	22,388	22,323	22,320
$D\ ^2\Pi_{3/2}$	23,074	22,852	22,790	22,734	22,671	22,673
$E\ ^2\Sigma_{1/2}$	25,616	25,509	25,554	25,544	25,501	25,520
$F\ ^2\Sigma_{1/2}$	28,410	28,067	28,080	28,050	27,995	28,019
$G\ ^2\Pi_{1/2}$	28,962	28,818	28,865	28,850	28,797	28,824
$G\ ^2\Pi_{3/2}$	29,391	29,276	29,323	29,309	29,257	29,284
$H\ ^2\Sigma_{1/2}$	29,860	29,654	29,697	29,681	29,629	29,663
$I\ ^2\Delta_{3/2}$	29,862	29,729	29,765	29,741	29,686	29,715
$I\ ^2\Delta_{5/2}$	30,006	29,868	29,904	29,878	29,824	29,852

SUPPLEMENTARY TABLE III. Theoretical results (in cm^{-1}) calculated at the FS-RCCSD-extAE3Z level for different numbers of correlated electrons. Unless specified in parenthesis, the shell contributions refer to electron shells belonging to the Ra atom.

State	97e	69e	35e	27e	17e	Shell contributions			
						$1s,...,3d$ (97e-69e)	$1s$(F) $4s4p4d4f$ (69e-35e)	$5s5p$ (35e-27e)	$5d$ (27e-17e)
$X\ ^2\Sigma_{1/2}$	0	0	0	0	0	0	0	0	0
$A\ ^2\Pi_{1/2}$	13,396	13,396	13,356	13,346	13,061	0	40	10	284
$B\ ^2\Delta_{3/2}$	14,604	14,604	14,528	14,491	14,133	0	76	37	358
$B\ ^2\Delta_{5/2}$	15,424	15,423	15,356	15,318	14,920	0	67	38	397
$A\ ^2\Pi_{3/2}$	15,476	15,475	15,423	15,400	15,084	2	51	24	316
$C\ ^2\Sigma_{1/2}$	16,792	16,790	16,745	16,727	16,446	2	45	18	280
$D\ ^2\Pi_{1/2}$	22,899	22,899	22,837	22,814	22,443	1	62	23	371
$D\ ^2\Pi_{3/2}$	23,238	23,237	23,177	23,154	22,769	1	60	23	385
$E\ ^2\Sigma_{1/2}$	25,582	25,582	25,542	25,536	25,208	0	40	7	328
$F\ ^2\Sigma_{1/2}$	28,457	28,457	28,400	28,389	27,995	0	57	11	394
$G\ ^2\Pi_{1/2}$	28,985	28,985	28,936	28,926	28,542	0	49	9	384
$G\ ^2\Pi_{3/2}$	29,400	29,400	29,351	29,339	28,955	1	49	11	384
$H\ ^2\Sigma_{1/2}$	30,009	30,009	29,960	29,950	29,565	0	49	10	385
$I\ ^2\Delta_{3/2}$	29,926	29,926	29,873	29,861	29,457	0	53	13	404
$I\ ^2\Delta_{5/2}$	30,078	30,078	30,026	30,013	29,605	0	52	13	408

SUPPLEMENTARY TABLE IV. Theoretical electronic excitation energies (in cm^{-1}) calculated with FS-RCCSD using the CV4Z and augmented CV4Z basis sets, for 27- and 69-electron correlation spaces. The results obtained with the non-augmented CV4Z basis set could not conclusively identify the energy of the three highest-lying states in this study.

State	27e-CV4Z	69e-CV4Z	69e-augCV4Z
$X\ ^2\Sigma_{1/2}$	0	0	0
$A\ ^2\Pi_{1/2}$	13,517	13,542	13,425
$B\ ^2\Delta_{3/2}$	15,009	15,079	14,770
$B\ ^2\Delta_{5/2}$	15,856	15,924	15,478
$A\ ^2\Pi_{3/2}$	15,648	15,690	15,549
$C\ ^2\Sigma_{1/2}$	17,079	17,112	16,773
$D\ ^2\Pi_{1/2}$	23,491	23,537	22,762
$D\ ^2\Pi_{3/2}$	23,819	23,864	23,105
$E\ ^2\Sigma_{1/2}$	26,052	26,069	25,739
$F\ ^2\Sigma_{1/2}$	29,149	29,185	28,671
$G\ ^2\Pi_{1/2}$	30,779	30,804	29,152
$G\ ^2\Pi_{3/2}$	31,362	31,390	29,580
$H\ ^2\Sigma_{1/2}$	—	—	30,105
$I\ ^2\Delta_{3/2}$	—	—	30,118
$I\ ^2\Delta_{5/2}$	—	—	30,268

SUPPLEMENTARY TABLE V. Calculated equilibrium bond length r_e, harmonic vibrational spacing $\omega_{\rm th}$, and equilibrium rotational constant $B_{\rm e,th}$ along with experimentally observed vibrational spacing $\omega_{\rm obs}$, rotational constant $B_{\rm 0,obs}$, spin-rotation coupling constant $\gamma_{\rm obs}$, and Λ-doubling constant $p_{\rm obs}$ for the states under 30,000 cm^{-1} from the ground state. All constants are in units of cm^{-1}. Information on the $X\ ^2\Sigma_{1/2}$ and $A\ ^2\Pi_{1/2}$ states is from Refs. [2, 3]. Not all known constants are shown for these two states. Details on the assignment and analysis of each state are shown in the subsection titled *State assignment*. The values for $p_{\rm pure}$ give the estimates from the pure precession approximation, used as a validity check. Conclusions on the structure of the excited states based on the molecular constants should be cautious of the limited power of contour fitting in determining B, γ, and p; rotationally resolved spectroscopy is necessary to precisely determine the constants. Unless otherwise noted by a subscript, errors in parentheses are statistical.

State	r_e (Å)	$\omega_{\rm th}$	$\omega_{\rm obs}$	$B_{\rm e,th}$	$B_{\rm 0,obs}$	$\gamma_{\rm obs}$	$p_{\rm obs}$	$p_{\rm pure}$
$X\ ^2\Sigma_{1/2}$	2.238(10)	440(4)	438.4(7)	0.1917(17)	$0.191985(5)_{\rm stat}(15)_{\rm syst}$	$0.00585(3)_{\rm stat}(7)_{\rm syst}$		
$A\ ^2\Pi_{1/2}$	2.244(10)	435(4)	432.2(7)	0.1910(17)	$0.191015(5)_{\rm stat}(15)_{\rm syst}$		$-0.41071(3)_{\rm stat}(7)_{\rm syst}$	-0.471
$B\ ^2\Delta_{3/2}$	2.255(10)	426(4)	425.9(34)	0.1892(17)	0.1896(14)			
$B\ ^2\Delta_{5/2}$	2.249(10)	430(4)	428.6(11)	0.1902(17)	0.1884(12)			
$A\ ^2\Pi_{3/2}$	2.240(10)	437(4)	444.2(9)	0.1917(17)	0.1911(6)			
$C\ ^2\Sigma_{1/2}$	2.259(10)	427(4)	424.4(3)	0.1885(17)	0.1886(4)	0.566(24)		
$D\ ^2\Pi_{1/2}$	2.235(10)	434(4)	431.1(4)	0.1926(17)	0.1922(4)		0.041(22)	0.049
$D\ ^2\Pi_{3/2}$	2.226(10)	444(4)	441.8(8)	0.1941(17)	0.1938(4)			
$E\ ^2\Sigma_{1/2}$	2.187(10)	481(5)	478.0(2)	0.2011(17)	0.1996(2)	0.070(2)		
$F\ ^2\Sigma_{1/2}$	2.172(10)	488(5)	491.7(18)	0.2039(17)	0.2053(24)	0.014(54)		
$G\ ^2\Pi_{1/2}$	2.180(10)	495(5)	496.2(6)	0.2024(17)	0.2007(22)		-0.360(42)	-0.407
$G\ ^2\Pi_{3/2}$	2.181(10)	496(5)	491.6(4)	0.2022(17)	0.2015(6)			
$H\ ^2\Sigma_{1/2}$	2.176(10)	510(5)		0.2031(17)	0.2108(20)	0.058(46)		
$I\ ^2\Delta_{3/2}$	2.178(10)	497(5)		0.2028(17)	0.2025(22)			
$I\ ^2\Delta_{5/2}$	2.178(10)	497(5)		0.2028(17)	0.2026(2)			

SUPPLEMENTARY TABLE VI. Observed spin-orbit (SO) interaction constants (in cm^{-1}) for the states in RaF assigned in this work ($A_{\rm RaF}$), and states in Ra^+ ($\zeta_{\rm Ra^+}$) whose valence configuration is most dominant in the composition of the corresponding RaF state.

	$A_{\rm RaF}$	$\zeta_{\rm Ra^+}$
$A\ ^2\Pi\ /\ 7p\pi$	2051(1)	3238
$B\ ^2\Delta\ /\ 6d\delta$	404(1)	663
$D\ ^2\Pi\ /\ 6d\pi$	362(1)	663
$G\ ^2\Pi\ /\ 8p\pi$	452(1)	1190
$I\ ^2\Delta\ /\ 7d\delta$	54(1)	198

SUPPLEMENTARY TABLE VII. Calculated potential energy curves (in cm^{-1}) as a function of internuclear distance (in Å) for the electronic states of the RaF molecule studied in this work. These data points were used to plot Suppl. Fig. 6.

R(Ra-F)	$X\ ^2\Sigma_{1/2}$	$A\ ^2\Pi_{1/2}$	$B\ ^2\Delta_{3/2}$	$A\ ^2\Pi_{3/2}$	$B\ ^2\Delta_{5/2}$	$C\ ^2\Sigma_{1/2}$	$D\ ^2\Pi_{1/2}$	$D\ ^2\Pi_{3/2}$	$E\ ^2\Sigma_{1/2}$	$F\ ^2\Sigma_{1/2}$	$G\ ^2\Pi_{1/2}$	$G\ ^2\Pi_{3/2}$	$I\ ^2\Delta_{3/2}$	$H\ ^2\Sigma_{1/2}$	$I\ ^2\Delta_{5/2}$
1.693	34097	47340	48994	49202	49727	51809	55405	55455	55971	57909	59743	60213	60510	61038	60640
1.852	13325	26782	28435	28582	29044	30906	35374	35464	36497	38609	39952	40420	40803	41099	40938
1.958	5995	19493	21034	21370	21688	23372	28377	28514	29953	32289	33302	33761	34208	34375	34347
2.011	3657	17157	18641	19071	19321	20923	26161	26326	27980	30443	31314	31766	32242	32364	32383
2.064	2002	15496	16923	17444	17630	19156	24601	24800	26674	29262	30014	30457	30960	31055	31103
2.117	908	14391	15762	16371	16496	17954	23580	23816	25915	28621	29281	29714	30241	30326	30386
2.170	274	13741	15057	15750	15817	17215	23000	23276	25604	28416	29009	29434	29980	30074	30128
2.223	16	13464	14725	15498	15511	16854	22778	23098	25655	28563	29110	29528	30092	30211	30243
2.238	0	13442	14687	15483	15481	16809	22770	23104	25730	28665	29201	29619	30188	30316	30339
2.275	60	13488	14695	15543	15507	16802	22845	23212	25999	28993	29510	29926	30505	30660	30659
2.328	348	13754	14907	15828	15745	16998	23145	23562	26575	29649	30147	30566	31159	31358	31314
2.646	4932	18208	19055	20335	20032	21125	27677	28432	32688	36181	36688	37186	37833	38256	38005
3.175	14833	27946	28369	30024	29521	30516	37405	38764	44306	48473	49452	50081	50768	50991	50965

SUPPLEMENTARY REFERENCES

[1] C. M. Western, PGOPHER: A program for simulating rotational, vibrational and electronic spectra, Journal of Quantitative Spectroscopy and Radiative Transfer **186**, 221 (2017).

[2] S. M. Udrescu, S. G. Wilkins, A. A. Breier, M. Athanasakis-Kaklamanakis, R. F. Garcia Ruiz, M. Au, I. Belošević, R. Berger, M. L. Bissell, C. L. Binnersley, A. J. Brinson, K. Chrysalidis, T. E. Cocolios, R. P. de Groote, A. Dorne, K. T. Flanagan, S. Franchoo, K. Gaul, S. Geldhof, T. F. Giesen, D. Hanstorp, R. Heinke, A. Koszorús, S. Kujanpää, L. Lalanne, G. Neyens, M. Nichols, H. A. Perrett, J. R. Reilly, S. Rothe, B. van den Borne, A. R. Vernon, Q. Wang, J. Wessolek, X. F. Yang, and C. Zülch, Precision spectroscopy and laser-cooling scheme of a radium-containing molecule, Nature Physics **20**, 202 (2024).

[3] R. F. Garcia Ruiz, R. Berger, J. Billowes, C. L. Binnersley, M. L. Bissell, A. A. Breier, A. J. Brinson, K. Crysalidis, T. E. Cocolios, B. S. Cooper, K. T. Flanagan, T. F. Giesen, R. P. de Groote, S. Franchoo, F. P. Gustafsson, T. A. Isaev, A. Koszorus, G. Neyens, H. A. Perrett, C. M. Ricketts, S. Rothe, L. Schweikhard, A. R. Vernon, K. D. A. Wendt, F. Weinholtz, S. G. Wilkins, and X. F. Yang, Spectroscopy of short-lived radioactive molecules, Nature **581**, 396 (2020).

[4] H. Lefebvre-Brion and R. W. Field, *The spectra and dynamics of diatomic molecules: revised and enlarged edition* (Elsevier, San Diego, CA, 2004).

[5] R. S. Mulliken and A. Christy, Lambda-Type Doubling and Electron Configurations in Diatomic Molecules, Physical Review **38**, 87 (1931).
